# Back to the Future: Lessons Learned in Modern Target-based and Whole-Cell Lead Optimization of Antimalarials

**DOI:** 10.2174/156802612799362977

**Published:** 2012-03

**Authors:** Arnab K Chatterjee, Bryan KS Yeung

**Affiliations:** 1 Genomics Institute of the Novartis Research Foundation 10675 John J. Hopkins Dr., San Diego, CA 92121, USA; 2 Novartis Institute for Tropical Diseases, 10 Biopolis Road, #05-01 Chromos, Singapore 138670

**Keywords:** Lead-optimization, malaria, whole-cell screening, spiroindolones, imidazolopiperazines.

## Abstract

Antimalarial drug discovery has historically benefited from the whole-cell (phenotypic) screening approach to identify lead molecules in the search for new drugs. However over the past two decades there has been a shift in the pharmaceutical industry to move away from whole-cell screening to target-based approaches. As part of a Wellcome Trust and Medicines for Malaria Venture (MMV) funded consortium to discover new blood-stage antimalarials, we used both approaches to identify new antimalarial chemotypes, two of which have progressed beyond the lead optimization phase and display excellent *in vivo *efficacy in mice. These two advanced series were identified through a cell-based optimization devoid of target information and in this review we summarize the advantages of this approach versus a target-based optimization. Although the each lead optimization required slightly different medicinal chemistry strategies, we observed some common issues across the different the scaffolds which could be applied to other cell based lead optimization programs.

## INTRODUCTION

The current arsenal of antimalarials represents drugs that are derived from both target-based and whole-cell approaches, such as dihydrofolate reductase inhibition (DHFR) and artemisinin, respectively. Target based approaches start with identification of an essential enzyme or pathway, ideally specific to the parasite. After the development of an *in vitro* biochemical assay, an HTS (high-throughput screen) can be executed to identify hit compounds. Subsequent screening on related human targets can be employed to determine selectivity. Although the advantage is that a selective compound can mitigate potential host toxicity, the reality is that selective compounds are rare in anti-infectives due to conserved target homology, target essentiality, and compound permeability among others. Moreover it can be difficult to predict the required selectivity index for low toxicity required for safety in man. Another common outcome often encountered in the early optimization in this approach is an *in vitro*/*in vivo* disconnect where excellent potency on the target does not translate to similar cellular activity or *in vivo* efficacy. More recently, the paucity of new drugs acting on “essential” targets has lead to a shift back to the traditional phenotypic screening approach. The advantage of this whole-cell screening approach is that although the mechanism of action is unknown, compounds identified from the screen can at least be deemed cidal and permeable. Recent reports of high throughput screening of chemical libraries on plasmodium have identified a number of new chemical entities many without a known mechanism of action (MOA) [1, 2, 14]. The lack of an established MOA, often considered a deficiency, has now become an opportunity with the advent of genomic techniques that can be used to identify new targets by using optimized compounds as probes [3]. The disadvantage of this approach however, is that the activity can also be the result of multiple pathways being targeted leading to very poor or narrow structure activity relationships (SAR) during the lead optimization phase (Table **1**).

A part of a search for new antimalarials we leveraged both approaches to identify new leads. Herein we summarize our efforts to date and conclude that although target-based approaches can lead to promising preclinical candidates, the whole-cell screening of high quality chemical libraries with support of early *in vitro* and *in vivo* PK profiling can identify a diverse range of active scaffolds resulting in shorter lead optimization timelines.

## THE TARGET BASED APPROACH

### Diaminoquinazolines for *Pf*DHFR

*Pf*DHFR is one of the few clinically validated targets in malaria and drugs such as pyrimethamine and cycloguanil have proven to be highly effective treatments in the antimalarial drug arsenal since their introduction. However after decades of use, widespread resistance has made these agents largely ineffective in endemic areas. The main driver of resistance is the result of multiple mutations in the enzymes' active site that effectively prohibit binding of the drug [4]. To identify new inhibitors with activity on *Pf*DHFR resistant strains, we profiled a known antifolate QN254 (**1**) which displayed equal potency on both wild type *Pf*DHFR and the highly pyrimethamine resistant *P. falciparum* V1S strain (Table **2**) [5]. Activity on the enzyme also translated well to cellular potency on wild type *P. falciparum* *in vitro* (EC_50_ = 9 nM). 

In addition, QN254 exhibited favorable oral pharmacokinetic properties in rodents and good efficacy against *Plasmodium berghei* in the murine model (Table **3**). Excellent *in vivo* oral activity (>99.99% reduction in parasitemia) was observed as low as 30 mg/kg with the average mouse survival prolongation out to 28.4 days. However at higher doses QN254 exhibited dose-limiting toxicity and six out of ten treated mice died around day ten despite being parasite free; the four surviving were cured and parasite free at day 30. We attributed this toxicity to activity on the closely related host DHFR enzyme. In humans, QN254 displays a 26-fold decrease in binding affinity on human versus *Pf*DHFR and has the lowest *K_i_* ratio when compared to cycloguanil and pyrimethamine. This was later confirmed in a 2-week rat toxicology study where QN254 was not tolerated upon repeated oral administration of greater than 50 mg/kg. Histopathological analysis revealed significant gastrointestinal and bone marrow toxicity. This type of toxicity on proliferative cells is consistent with on-target effects of sustained DHFR inhibition and underscores the importance of enzyme selectivity towards establishing an adequate therapeutic index [6, 7].

Despite the fact that the *in vitro* selectivity is known for cycloguanil and pyrimethamine and that they are well tolerated drugs, it remain unclear what the true selectivity index would be for safety with the next generation anti-folates.

### Purines for *Pf*CDPK1

In *P. falciparum*, calcium-dependent protein kinase 1 (*Pf*CDPK1) is expressed during schizogony in the blood and sporozoite stages and is ultimately required for parasite invasion of host cells, parasite motility, and potentially cytokinesis [8]. We identified a series of purine-based, ATP competitive kinase inhibitors from a target-based screen of approximately 20,000 compounds on recombinant *Pf*CDPK1 [9]. Of the most promising hits, the 2,6,9-trisubstitutes purines made up the largest class of compounds coming out of the screen and were therefore chosen as a lead series for chemical optimization. The main challenge for this series proved to be obtaining an interpretable correlation between enzyme inhibition and cell-based activity. 

Within the hit series, compound **2** (purfalcamine) was found to have favorable potency in *in vitro* biochemical assays, whole cell proliferation and *in vivo* parasitemia suppression, suggesting that the compound may be acting on PfCDPK1 within the parasite to exert these effects. Although other chemical modifications on the purine ring were incorporated during the optimization, substitutions on the *N*-9 phenyl generally included the most potent enzyme inhibitors (Table **4**). *m*-Fluoro- (**2**) and the carboxy substituted derivatives **6** and **7** proved to be the most optimal substitutions and the best enzyme inhibitors, however improving enzyme activity did not lead to improved cellular activity. For example despite a greater than 200-fold improvement in enzyme potency (**2** vs. **3**), the cellular activity was similar. This may suggest that cell permeability was reduced in compound **2** relative to compound **3 **or that there were other cellular targets present with compound **3**. Poor compound permeability might also explain the complete loss of activity at the cellular level for carboxy derivatives **6** and **7**. The overall SAR we observed for this series were typical of compounds **4** and **5** where poor enzyme activity and moderate cellular potency were observed.

We were ultimately unable to break the 10 nM barrier for enzymatic activity or completely rule out off-target activity on other intracellular targets since alternate drivers of *in vitro* potency have been previously described for other purine templates [10]. We interpreted the poor kinase selectivity for *Pf*CDPK1 over human kinases to rationalize the moderate cytotoxicity observed. The EC_50_ value for compound **2** on *P. falciparum* was 230 nM, giving a therapeutic window ranging from 23-fold to 36-fold on mammalian cell lines (EC_50_s for CHO = 12.3 µM, HEp2 = 7.2 µM, HeLa = 7.0 µM, and Huh7 = 5.5 µM). Based on this we surmised that increasing enzyme activity, without being able to address selectivity would further reduce the safety index. In addition to the lack of kinase selectivity, the series as a whole displayed poor physicochemical properties making it difficult to improve the pharmacokinetic (PK) properties of the lead compound towards an acceptable oral bioavailability required for an anti-malarial drug [11].

Our experience and the experiences of others with target based lead optimization does not preclude this approach [12]. Successes with *Pf*DHFR and *Pf*DHPS have made a profound impact on malaria chemotherapy and more recently triazolopyrimidines acting on *P. falciparum* dihydroorotate dehydrogenase (*Pf*DHODH) are currently in preclinical development towards proof of concept in man [13].

Any chemical series acting on a parasite target needs to display SAR which reflects not only potent enzyme and cellular activity but also selectivity towards the host enzyme, if present. As an excellent safety and tolerability profile are critical for development of next generation antimalarials, achieving optimal enzyme selectivity early will help mitigate potential adverse effects. Given that we were unable to achieve whole-cell activity and/or selectivity in the above chemical series, we directed our efforts towards the historically more successful cell-based approach. When coupled with a novel whole-cell HTS of large compound libraries, we were able to identify several new chemotypes for medicinal chemistry optimization.

## THE WHOLE-CELL BASED APPROACH

We screened both our in-house compound collection (~2.3M compounds) and natural products library (12,000 compounds) using a cell based *Plasmodium falciparum* proliferation assay [14]. This screen provided data on a large number of chemically diverse anti-malarial compounds. These nearly 5700 hits have been deposited into the public EBI database by GNF/Novartis [15]. A number of these hits originated from kinase-directed libraries and many of which are pan-kinase inhibitors. De-convoluting and eliminating inherent kinase activity with many of these scaffolds while maintaining cellular potency was the first step in the hit-to-lead phase for these particular chemotypes. What follows is a summary of the chemical optimization of some kinase-like and non-kinase scaffolds which came out of the whole-cell screening effort.

## BENZAMIDES

The significant disconnect from enzyme to cellular potency with broad-kinase based scaffolds (such as *Pf*CDPK1 described above) led us to ask a different question: Can cellular potency, namely antimalarial activity, be disconnected from the pharmacophore responsible for kinase activity? Eliminating the kinase pharmacophore early on in lead optimization would also reduce molecular weight, improve ligand efficiency, and potentially reduce off-target toxicity [16, 17].

Compound **8**, originally designed as a pan-kinase Bcr-Abl inhibitor, displays moderate activity against the chloroquine sensitive 3D7 strain of *P. falciparum* Fig. (**1**) [18]. A scan of solubilizing groups quickly identified **9** with nearly a three fold improved cellular potency. Small changes on the western phenyl ring were also tolerated and lead to an equipotent compound (**10**). More importantly this change led to a complete loss of human kinase activity as measured in a Ba/F3 cell line RTK panel [19]. From this observation we concluded that the pharmacophore responsible for kinase activity might not be essential for anti-malarial activity, and thus removing this moiety from the scaffold could represent the minimum pharmacophore [20]. As expected the loss of activity on human kinases also resulted in a better safety profile of the series on various mammalian cell lines (CC_50_s > 10 µM). This reinforced our conclusion that the kinase-binding functionality in the purines for *Pf*CDPK1 was likely driving the inherent cell toxicity observed for that series.

During the optimization of the benzamide series, we also observed a difference in activity between the chloroquine sensitive 3D7 strain and the multi-drug resistant W2 strain. The piperidinyl-pyrrolidine functionality on compound **11** displayed the largest shift in potency between 3D7 and W2 strains (nearly six-fold), however we observed that a minute structural change from the piperidinyl-pyrrolidine system to the *bis*-piperidine system (**12**-**15**) improved activity on the W2 strain across several compounds (Table **2**). Unfortunately we were unable to further exploit the incremental activity gains of the *bis*-piperidines and compounds remained two- to four-fold less active on drug resistant strains. Moreover when compound **14** was screened against fifteen different *P. falciparum* wild type and drug resistant strains, the *in vitro* EC_50_s hovered between 100-300 nM. Based on this and compared to known antimalarials, we predicted this series lacked the *in vitro* potency required for *in vivo* efficacy.

In addition to the eastern part of the molecule there was a significant effort to optimize the central CF_3_-phenyl and western benzamide moieties. Replacement of either central phenyl ring with other heterocycles or attempts to replace the amide bond (e.g. sulfonamide and urea linkages) consistently resulted in a loss of *in vitro* potency. Overall the benzamide series was unable to achieve the low nanomolar potencies required for blood-stage efficacy and when combined with the narrow SAR we found it difficult to progress the compounds forward. Despite this we concluded that hit compounds representative of kinase inhibitors can be useful starting points for lead optimization providing that the intrinsic kinase activity can be separated from cellular activity early on.

## IMIDAZOLOPYRIMIDINES

The imidazolopyrimidines were another class of anti-malarial hits previous optimized from a human kinase program. The hit compound (**16**) possessed moderate activity on the 3D7 parasite strain and did not exhibit significant activity against a panel of 40 mammalian kinases, which suggested it was not a pan-kinase inhibitor [21]. The primary liability of compound **16** however was the low aqueous solubility (36 µM at pH 6.8) and likely driven by the high logD of the compound Fig. (**2**). We primarily sought to address this by the introduction of hydrophilic or ionizable groups onto the scaffold. A scan of the optimal substitution sites on the molecule identified C-2 on the central pyrimidine as the best position. To further explore this site we generated a focused library which identified the *N*-methylpiperidine derivative (**17**), which displayed an EC_50_ of 34 nM on 3D7. Compound **17** was also evaluated against a panel of fifteen drug resistant strains of *P. falciparum*. EC_50_ values across all strains were lower than 140 nM, many of which were below 100 nM suggesting that the compound might be a good lead to address drug resistance.

The incorporation of the basic functionality on the central ring in close proximity to the presumed kinase binding pharmacophore also maintained a clean human kinase profile when tested on 190 kinases in both biochemical and cellular assay formats. Unfortunately despite the greater than 10-fold improvement in potency and the addition of an ionizable group, both the aqueous solubility and the clogD were essentially unimproved in **17** over the hit compound and also introduced a potential hERG channel blocker pharmacophore by incorporation of a basic/lipophilic group [22]. The fact that we were unable to improve physiochemical properties and *in vitro* potency in the same molecule from the hit, we decided to investigate other scaffolds.

## IMIDAZOLOPIPERAZINES

The imidazolopiperazines represent the latest scaffold we have optimized from the whole-cell screen which resulted in the identification of a new chemotype with promising blood-stage activity [23]. Imidazolopiperazine hits from the screen (**18a-c**) represented an attractive series displaying favorable potency on both drug-susceptible and -resistant strains, while maintaining good selectivity Huh7 cells. The selectivity window of cytotoxicity was markedly better than those of the kinase directed scaffolds described above. Compound **18a** displayed excellent aqueous solubility (>175 µM at pH 6.8) and was clean on a panel of cytochrome P450 isoforms (IC_50_ >10 µM). Early issues associated with the scaffold included metabolic instability and a slight hERG signal in a binding assay (IC_50_ = 19 µM).

There were several key concepts that aided the progression of this series outside of the normal broad-based SAR to improve potency. First, in the early SAR development we identified potential metabolic liabilities on the scaffold (in silico) and quickly moved to address them without always waiting for microsomal stability data. This type of intuitive approach allowed us to replace the methylene dioxyphenyl group which was identified to be prone to aromatic oxidation and dealkylation in other chemical series [24]. The unsubstituted phenyl ring at the 2-position of the imidazolopiperazine was also identified as another potential area of metabolic oxidation and was modified very early by the introduction of a fluorine atom at the para-position Fig. (**3**). 

The early toxicity profiling also identified a potential hERG liability which we associated with the primary amine of the glycine moiety. A significant effort was spent on understanding the SAR of the hERG activity and differentiating it from *in vitro* potency. As the terminal amine was required for both potency and favorable PK properties, we found that original glycine turned out to be the optimal substituent in terms of lower hERG activity. In addition to improving potency and understanding the SAR of the hit, we extensively profiled early derivatives in a number of *in vitro* and *in vivo* PK assays including snapshot PK experiments in mice as an early assessment of oral exposure [25]. 

We also found that an *in vitro* permeability assay (PAMPA), metabolic stability in liver microsomes and mouse snapshot oral PK were predictive in helping to understanding efficacy in the mouse model. Compounds were prioritized that showed good permeability and low clearance in liver microsomes. Access to these types of *in vitro* assays, both within industry and academia, can greatly accelerate the drug discovery process.

Compounds **19** and **20** were tested in the *P. berghei* mouse model to assess how *in vitro* activity translated to *in vivo* efficacy (Table **7**) [26]. At all dosing regimens both compounds showed excellent activity, reducing parasitemia by more than 99%. Despite the excellent activity, clear differences in the survival of the mice were observed. At a low dose of 30 mg/kg, and despite the greater than 99% activity, these compounds were unable to prolong mouse survival much past the control group. However increasing the dose to 100 mg/kg or dosing for three consecutive days (3 x 30 mg/kg) prolonged mice survival for about two weeks. Both **19** and **20** showed parasitemia reduction activities and survival prolongation comparable those of chloroquine (CQ) and artesunate (AS) at similar doses.

The imidazolopiperazines as a class showed a number of key differences from the other cellular hits described above. Although establishing SAR towards low nanomolar potency is critical early on, understanding the drivers of good PK is also needed to establish an *in vitro*/*in vivo* correlation as early as possible. This correlation ultimately provides confidence in the quality of the hit to warrant further development. The optimization of **18a** from hit to lead (**19** and **20**) provided a basis for a successful lead optimization campaign towards a preclinical candidate. 

## SPIROINDOLONES

Natural products or drugs derived from natural products not only represent the mainstay of anti-malarial chemotherapies but also the successful implementation of whole-cell screening approaches [27]. Based on this premise we screened the Novartis natural product library containing both pure natural products and natural product-like synthetic compounds. The hit compound (**21**) was identified as a racemic mixture of unknown configuration and displayed moderate potency on both on the drug susceptible NF54 and chloroquine-resistant K1 *P. falciparum* strain Fig. (**4**) [28]. The presence of the central spirocenter gave the molecule a unique overall shape compared to the achiral, planar hits often encountered in HTS screens and distinguished itself from some of the other natural product hits which were high molecular weight complex structures. The compound displayed moderate aqueous solubility (75 µM at pH 6.8) and 99% permeability in a PAMPA assay. This *in vitro* PK profile correlated well with a full *in vivo* PK experiment in mouse where a single 25 mg/kg oral dose resulted in a high C_max_ (1.4 µM), good oral bioavailability (F = 59%), and an oral T_1/2 _of nearly four hours. Compound **21** also had a favorable early safety profile when screened against a panel of mammalian cell lines, hERG, cytochrome P450 isoforms, and in a panel of human relevant proteins (IC_50_s >10-30 µM).

One of our first tasks was to de-convolute the activity of the racemate. This was achieved by chiral separation followed by an X-ray crystal structure to unambiguously elucidate the configuration of both stereocenters. Interestingly we also observed a significant difference in the activity of compounds **22** and **23**, with only the 1*R*,3*S* enantiomer responsible for biological activity. 

Based on the favorable physicochemical properties and *in vivo* PK of **21**, we elected to evaluate it in the *P. berghei* mouse model. Remarkably, a single oral dose of 100 mg/kg resulted in a 96% reduction in parasitemia. Although only a slight prolongation in mouse survival over control (seven days) was observed at this dose, it was nonetheless an unexpected result given the moderate *in vitro* potency. We surmised that *in vivo* efficacy might be improved upon increasing the potency of the compound. 

Differences in the *in vitro* potency of enantiomers extended to other properties as well; most notably metabolic stability in the presence of liver microsomes. For example compound **24** (six membered spiro) displayed improved *in vitro* potency over the hit and moderate clearance in mouse and human microsomes (Table **8**). However profiling the individual enantiomers separately showed that the active enantiomer (**26**) was metabolized much more readily than the inactive enantiomer (**25**). Based on this result our task was to combine the low clearance of the inactive enantiomer with the potency of the active enantiomer. We identified the aromatic portion of the tetrahydro-β-carboline ring on **26** as metabolically susceptible and focused on introducing substituents to hinder oxidation at these sites. 

The microsomal clearance assay proved to be predictive in assessing the metabolic stability of the spiroindolones; and those derivatives which displayed high stability were also found to have low clearance in mouse PK experiments. By systematic substitutions on the indole moiety of the spiroindolones we found that blocking the C-7 position had the most marked effect on increasing the half life in the presence of liver microsomes (Table **9**). Fortuitously indole substitutions also provided 3-10 fold gains in potency, with the 6,7-di-substituted derivatives displaying the most additive effects (**29** and **30**).

The improvements in the metabolic stability and potency of the spiroindolones translated remarkably well to *in vivo* efficacy in the *P. berghei* infected mouse model and spiroindolones **26**, **29**, and **30** out performed current antimalarial treatments under similar dosing regimens (Table **10**). Single oral doses of 30 mg/kg showed survival prolongation in mice greater than controls; out to 10 days to two weeks. Multiple daily doses of 3 x 30 mg/kg yielded more striking results with mouse survival out to 18.8 and 23.8 days with compounds **26** and **29**, respectively. The same dosing regimen with **30** prolonged survival out to 29.1 days in a 30 day experiment and single oral dose of **30** at 100 mg/kg completely cleared a **P. berghei** infection in all treated mice [29]. Orally administered **30** displayed a long half-life (*T*_1/2_ = 10 hours) and excellent bioavailability (*F* =100%) in mice, consistent with a low dose, long lasting antimalarial drug.

Retrospectively compound **21** represented a high quality hit with favorable physicochemical properties and good oral PK properties. The spirocenter imparted a three dimensionality to the overall shape of the molecule which we believe gave the molecule good intrinsic solubility not generally encountered in highly planar, aromatic compounds. Although part of our natural product collection, **21** is not a natural product and instead is fully synthetic [30]. Finally, compound **21** also happened to represent the minimum pharmacophore of the series. Nearly all of the essential structural components required for potency in the optimized compound can be found in the hit (e.g. the spirocenter, 3-methyl substituent, and the substituted oxindole moiety). We believe this is another one of the key reasons we were able to rapidly and efficiently optimize the series [28].

## CONCLUSION

There are numerous examples of successful antimalarial drugs derived from natural products where the target and mode of action was unknown at the time, most notably quinine and artemisinin. During the discovery and development of these drugs, the whole-cell approach was the only method available to drug discovery. More recently, armed with the genome of the most deadly malaria parasite, *P. falciparum*, new target based approaches were predicted to provide an influx of next generation antimalarials working through new, parasite specific targets. Unfortunately this has yet to be realized as many genetically validated targets exist but few have been chemically and more importantly, clinically validated.

Although target-based approaches in the search for new antimalarials remains an active area of research, new chemical entities from HTS whole-cell screens of large chemical libraries are beginning to bolster the antimalarial pipeline at a more efficient pace than target-based screening. Cell-based screening has provided a large amount of chemical diversity but the subsequent triaging and prioritization of compounds can be challenging. Compound prioritization should be assessed not only by potency but also by physicochemical properties and supported by early *in vivo* PK experiments. By identifying hit scaffolds which have favorable intrinsic properties such as good solubility and permeability, or by showing that the scaffold can tolerate the introduction of new structural elements which impart such favorable properties, may help simplify the optimization or at a minimum serve to reach a no-go decision sooner.

Of the scaffolds described above, only the spiroindolones and imidazolopiperazines were able to progress past lead optimization towards preclinical development. In retrospect one crucial aspect that both compound series shared was the early establishment of an *in vitro*/*in vivo* correlation with good physiochemical properties. Another important detail was that the original hit or lead compound was very closely related to the minimal pharmacophore required for antimalarial activity and thus required no gross changes, again simplifying the lead optimization. These attributes were clearly lacking in the other scaffolds discussed above and required a much more lengthy optimization campaign. Hopefully, these strategies and lessons learned can assist in optimization of the many unexplored hits that are now in the public domain for the betterment of malaria patients.

## Figures and Tables

**Fig. (1):**
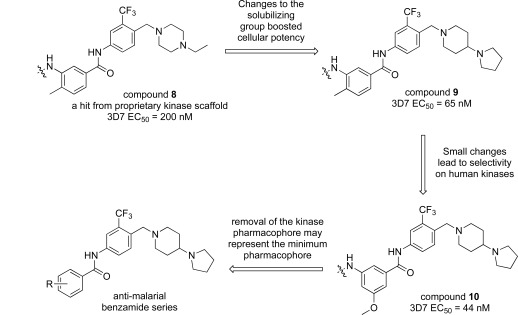
Substituted benzamides derived from a kinase pharmacophore.

**Fig. (2):**
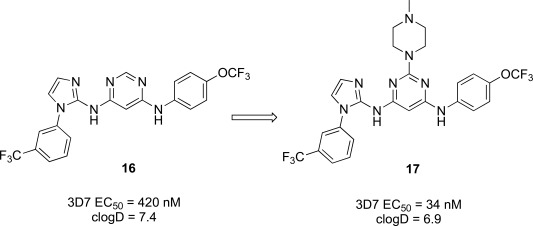
Optimization path for imidazolopyrimidine kinase library hit.

**Fig. (3):**
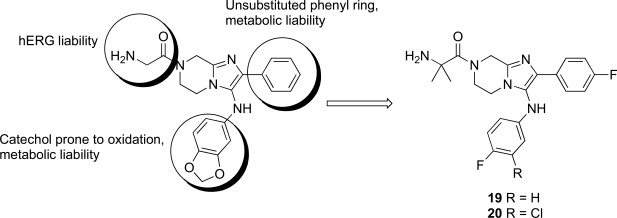
Summary of hit to lead for the imidazolopiperazines series.

**Fig. (4):**
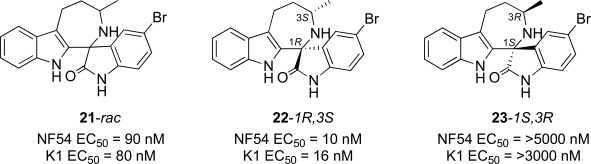
The activity of the spiroindolone hit and its stereoisomers.

**Table 1.** The General Sequence of Two Approaches Used to Develop New Antimalarial Candidates

Target-based Approach		Cell-based Approach	
Target identification from genomic data of the parasite Genetic validation of the molecular target Target expression, assay development High-throughput screening for hits against the target Lead optimization of hits	**Pros:** Known mechanism of action; amenable to structure-based lead optimization **Cons:** Protein expression and assay development can be challenging; good inhibitor does not mean good drug	Identify libraries or classes of molecules with potential biological activity on the parasite High-throughput screening for hits against on the whole parasite Lead optimization of hits	**Pros:** Active on plasmodium (cell penetration); genomic tools can lead to the identification of new targets in parallel **Cons:** Mechanism of action unknown; lead optimization can be challenging (presence of multiple targets)

**Table 2.** Selectivity and Binding of QN254 on *Pf*DHFR and hDHFR

Compound	*K_i_* (nM)			*K_i_* Ratio
	WT-*Pf*DHFR^a^	QM-*Pf*DHFR^b^	*h*DHFR^c^	*h/Pf*-DHFR
Cycloguanil	1.51 ± 0.1	454 ± 38	55.6 ± 7.8	37
Pyrimethamine	0.59 ± 0.05	385 ± 163	30.8 ±1.4	52
QN254	0.39 ± 0.05	0.58 ± 0.06	10.2 ± 0.6	26

a wild type *Pf*DHFR

b quadruple mutant *Pf*DHFR,

c human DHFR.

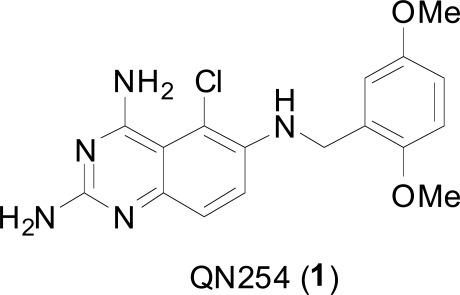

**Table 3.** Antimalarial Activity of QN254 in the *P. berghei* Murine Model^a^

Dosing Regimen	Parasitemia Reduction (%)	Average Survival (days)	Cure Rate (%)	Toxicity Rate (%)
3 x 30	> 99.99	28.4	80	0
3 x 60	> 99.99	Not available	60	40^b^
3 x 100	> 99.99	Not available	20	80^b^

a Percent inhibition of parasitemia compared to untreated controls. The mean survival of control animals was 5.6 to 6.2 days

b Mice died around day 10 and were parasite free.

**Table 4.** Activity of Purines on PfCDPK1 and *P. falciparum* 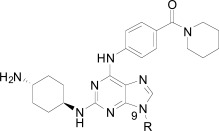

Compound	R	*Pf*CDPK1 IC_50_ (nM)^a^	*P. falciparum* 3D7 EC_50_ (nM)^a^
2	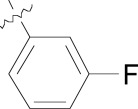	17	230
3	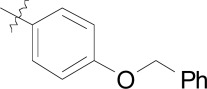	4040	312
4	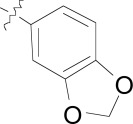	342	400
5	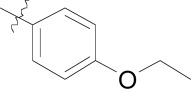	240	535
6	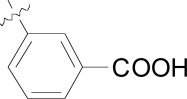	71	> 10,000
7	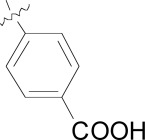	28	> 10,000

a Values are mean of two experiments. Each assay plate has mefloquine, sulfadoxine and artemisinin as internal standards. The EC_50_ values for standard compounds match literature values.

**Table 5.** The Activity of Piperidyl Benzamides on Drug Resistant Strains 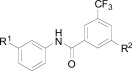

Compound	R^1^	R^2^	*P. falciparum* 3D7 EC_50_ (µM)^a^	*P. falciparum* W2 EC_50_ (µM)^a^
11	Cl	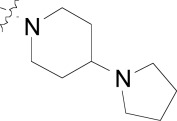	0.134 ± 0.056	0.888 ± 0.112
12	Cl	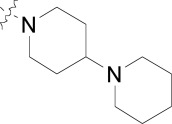	0.097 ± 0.018	0.276 ± 0.014
13	CF_3_	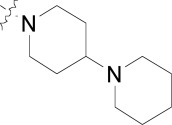	0.149	0.299
14	Cl	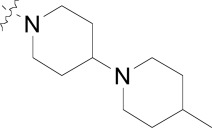	0.140	0.323
15	CF_3_	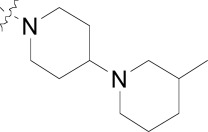	0.058	0.211

a Values are mean of two experiments. Each assay plate has mefloquine, sulfadoxine and artemisinin as internal standards. The EC_50_ values for standard compounds match literature values.

**Table 6.** The Activity of Imidazolopiperazine Hits 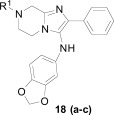

Compound	R^1^	*P. falciparum* 3D7 EC_50_ (nM)^a^	*P. falciparum* W2 EC_50_ (nM)^a^	Cytotoxicity (µM) Huh7^a^
18a	Gly	63	97	> 10
18b	(DL)-Phe	235	271	> 10
18c	(DL)-Leu	116	119	> 10

a Values are mean of two experiments.

**Table 7.** *In Vivo* Efficacy of Imidazolopiperazine Leads ^a^

Compound	1 x 30 mg/kg		1 x 100 mg/kg		3 x 30 mg/kg	
	Activity (%)	Survival (Days)	Activity (%)	Survival (Days)	Activity (%)	Survival (Days)
19	99.3	7.7	99.6	13.3	99.9	14.0
20^b^	99.4	7.7	99.4	17.0	99.8	17.7
CQ	99.7	8.7	>99.9	12	98.6	18.8
AS	89	7.2	97	6.7	98	7.2

a average parasitemia reduction; survival of 6-7 days for untreated mice; 7% Tween 80/3% ethanol formulation.

b 75% PEG300/25%D5W formulation.

**Table 8.** Differences in Potency between Enantiomers Extended to Metabolic Stability

Compound	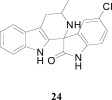		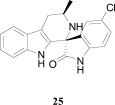		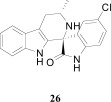	
NF54 EC_50_ (nM)	27		>5000		9.2	
Liver Microsomesa^a^	CL_int_	*t*_1/2_ (min)	CL_int_	*t*_1/2_ (min)	CL_int_	*t*_1/2 _(min)
Mouse	Med	26.5	Low	103	High	1.8
Human	Med	9.9	Low	95	High	1.2

a intrinsic clearance in liver microsomes; where high clearance corresponds to low stability in the presence of liver enzymes

**Table 9.** Additive SAR Simplified the Lead Optimization of the Spiroindolones 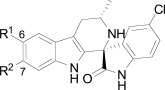

Compound	R^1^ = F R^2^ = H 27		R^1^ = H R^2^ = F 28		R^1^ = F R^2^ = F 29		R^1^ = F R^2^ = Cl 30 (NITD609)	
NF54 EC_50_ (nM)	3.4		3.5		0.2		0.9	
Liver Microsomes^a^	CL_int_	*t*_1/2_ (min)	CL_int_	*t*_1/2_ (min)	CL_int_	*t*_1/2_ (min)	CL_int_	*t*_1/2_ (min)
Mouse	High	4.2	Low	53	Low	49.1	Low	49
Human	Med	10	Low	53.3	Low	56.4	Low	76

a intrinsic clearance in liver microsomes; where high clearance corresponds to low stability in the presence of liver enzymes.

**Table 10.** *In Vivo* Efficacy of the Optimized Spiroindolones ^a^

Compound	1 x 30 mg/kg			3 x 30 mg/kg		
	Activity (%)	Survival (Days)	Cure Rate (%)	Activity (%)	Survival (Days)	Cure Rate (%)
26	99.9	10.7	0	99.9	18.8	60
29	99.6	12.0	0	99.8	23.8	80
30^b^	99.6	13.3	0	99.8	29.1	90
CQ	99.7	8.7	0	99.9	14.0	0
AS	92.2	7.3	0	99.0	11.8	0

a average parasitemia reduction; survival of 6-7 days for untreated mice; cure was no parasites at day 30; compounds were formulated in 10% ethanol, 30% PEG400, 6% vitamin E TPGS;

b formulated in 0.5% MCM / 0.1% Solutol HS15.
